# The molecular crosstalk between RECQ helicases and associated proteins in human genetic disorders

**DOI:** 10.1016/j.jgeb.2026.100824

**Published:** 2026-09-29

**Authors:** Yashaswini Reddy, Gargi Joshi, Swechha Mahapatra, Ipshita Das, Babitha Kampa Sundara

**Affiliations:** a Department of Biophysics, Manipal School of Life Sciences, Manipal Academy of Higher Education, Manipal, India; b Manipal School of Life Sciences, Manipal Academy of Higher Education, Manipal, India

**Keywords:** BLM, cancer predisposition, Genomic instability, RECQ helicase, WRN

## Abstract

RECQ helicases involve a family of DNA unwinding enzymes, namely, RECQ1, Bloom syndrome protein, Werner syndrome protein, RECQL4, and RECQ5. These proteins interact with various proteins to form complexes and carry out a range of physiological functions. These genes are involved primarily in molecular pathways such as DNA replication, DNA repair, homologous recombination and telomere maintenance. Dysfunction of these protein complexes leads to chromosomal instability and impaired DNA repair and replication, thus increasing susceptibility to tumor formation. Bloom syndrome protein helicase is characterized by its interaction with proteins such as topoisomerase IIIα and RecQ-mediated genome instability, which prevents excessive sister chromatid exchanges. Werner syndrome protein helicases work in association with enzymes such as Flap Endonuclease 1 and Replication Protein A to maintain telomeric length and resolve secondary structures. RECQL4 helicase acts as a versatile enzyme while initiating DNA replication, carrying out DNA damage repair and contributing to the maintenance of mitochondrial function. The functions and genetic implications of the RECQ1 and RECQ5 helicases have not yet been well established; however, their involvement in DNA repair pathways has been studied. This review outlines the major genetic disorders caused by mutations in the Bloom syndrome protein, Werner syndrome protein and RECQL4 genes, which are associated with Bloom's syndrome, Werner's syndrome and Rothmund-Thomson syndrome, among others. In addition, it explores the roles of RECQ helicases, their molecular interactions in major pathways and their potential as therapeutic targets.

## Graphical abstract

**Figure:**
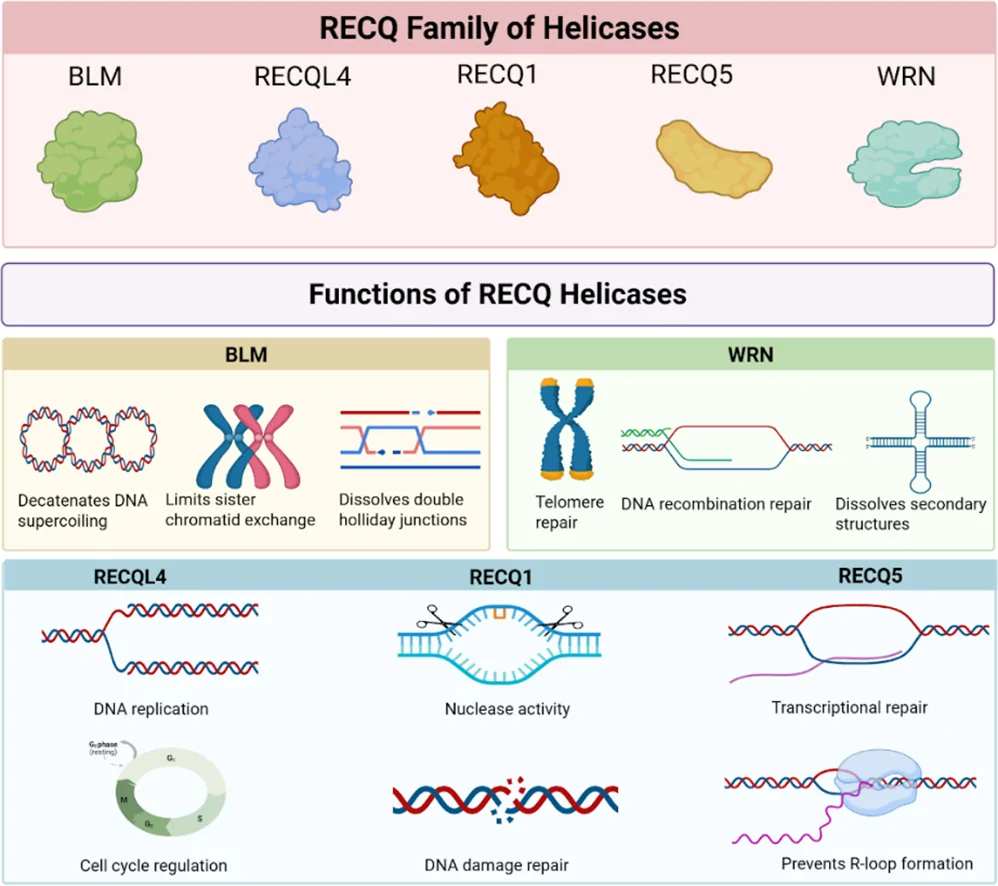

## Abbreviation table

**Table:** 

ALT	Alternative Lengthening of Telomeres
ANAPC1	Anaphase Promoting Complex Subunit 1
ATRX	Alpha Thalassemia/Mental Retardation Syndrome X-Linked
AURKB	Aurora B Kinase
BGS	Baller-Gerold Syndrome
BLM	Bloom syndrome protein
BRAFT	BRCA1-Associated FANCD2 and RAD51 Tumor suppressors complex
BrdU	Bromodeoxyuridine
BS	Bloom's Syndrome
BTR	BLM-TOPIIIα-RMI
CK2	Casein Kinase 2
CPT	Camptothecin
CR1	Conserved Region 1
CR2	Conserved Region 2
DSB	Double-Strand Break
ETOP	Etoposide
EXO1	Exonuclease1
FA	Fanconi Anemia
FEN1	Flap Endonuclease 1
HCC	Hepatocellular Carcinoma
HJ	Holliday Junction
HR	Homologous Recombination
hTOPIIIα	Human Topoisomerase IIIα
ICL	Intercellular Cross-Linking
iPSCs	Induced Pluripotent Stem Cells
MM1	Myc-Max 1
MM2	Myc-Max 2
MRC 1	Mitochondrial Respiratory Complex I
MSI	Microsatellite Instability
mtDNA	Mitochondrial DNA
NCO	Non-Crossing Over
OXPHOS	Oxidative Phosphorylation
PARP1	Poly (ADP-Ribose) Polymerase 1
PCNA	Proliferating Cell Nuclear Antigen
PCR	Polymerase Chain Reaction
PIAS1	Protein Inhibitor of Activated STAT 1
RAD51	RADiation Sensitive 51
RBP1	Retinol Binding Protein 1
RMI	RecQ-Mediated genome Instability
RNAPI	RNA Polymerase I
RNAPII	RNA Polymerase II
ROS	Reactive Oxygen Species
RPA	Replication Protein A
RTS	Rothmund-Thomson Syndrome
SCE	Sister Chromatid Exchange
SDSA	Synthesis-Dependent Strand Annealing
SRSF1	Serine/Arginine-Rich Splicing Factor
ssDNA	Single-Stranded DNA
TCR	Transcription-coupled DNA Repair
TOPIIIα	Topoisomerase IIIα
TOPO I	Topoisomerase I
TOPO II	Topoisomerase II
TRF2	Telomeric Repeat-binding Factor 2
WH	Winged Helix
WRN	Werner syndrome protein
WS	Werner Syndrome
ZBD	Zinc Binding Domain

## 1 Introduction

RECQ family helicases are evolutionarily conserved proteins found from bacteria to humans that play a vital role in DNA damage repair and preserving genomic integrity. RECQ helicases are key enzymes in multiple double-strand break (DSB) repair pathways and have evolved to preserve DNA structure and integrity.^1^ They also contribute to the replication checkpoint as regulators, sensors, and adaptors and are crucial for cell survival.^2^ In humans, there are five helicases belonging to this family: RECQ1, Bloom syndrome protein (BLM), Werner syndrome protein (WRN), RECQL4, and RECQ5.

RECQ1, the first identified human RECQ homologue, is located on chromosome 12p12 and encodes a 649-amino-acid protein. It comprises four distinct domains: the N-terminus, helicase domain, RECQ-specific C-terminal domain, and C-terminus.^3^ The RECQ1 helicase is involved in transcription, the cell cycle, telomere maintenance, and DNA repair processes and is also integral for Holliday junction (HJ) unwinding and protein oligomerization.^4^ The BLM helicase is encoded by the BLM gene, which is located on human chromosome 15q26.1. This protein consists of multiple domains, including an N-terminal domain, a helicase domain, an RECQ-dependent C-terminal domain, and an RNase-D-C-terminal domain. It is a 1417-amino acid, ATP-dependent 3′-5′ DNA helicase that interacts with various proteins, such as topoisomerase IIIα (TOPIIIα) and the RecQ-mediated genome instability (RMI) complex. These interactions lead to the formation of protein complexes that play crucial roles in preserving genomic stability by resolving replication intermediates and preventing unwanted genetic recombination.^5^ The WRN gene, positioned on human chromosome 8p12, comprises 34 coding exons and encodes the WRN protein. This protein features an ATP-dependent 3′-5′ helicase domain in its central region, a 3′-5′ exonuclease domain at the N-terminus, and a nuclear localization sequence at the C-terminus.^6^ The WRN gene is unique to the RECQ family because of its nuclease activity. WRN helicases can interact with various proteins, including DNA topoisomerase I (TOPO I), replication protein A (RPA) and flap endonuclease 1 (FEN1), to carry out a wide range of functions, including unwinding dsDNA, resolving DNA structures such as G4 quadruplexes and thus resuming stalled replication forks.^7^ The RECQL4 helicase is encoded by the RECQL4 gene, which is present on chromosome 8q24.3 and has 21 exons. It contains a characteristic RECQ-helicase domain along with its N-terminal and C-terminal domains.^8^ The expression of RECQL4 mRNA varies depending on the tissue type and cell cycle stage, and it usually peaks during the S phase. RECQL4 localizes primarily in the nucleoplasm inside the nucleus, and a fraction of it colocalizes in telomeres and mitochondria. The N-terminus of the protein plays a crucial role in DNA replication; however, the C-terminus has not yet been well characterized.^9^ The RECQ5 gene is located on chromosome 17q25.1. RECQ5 is a nucleus-localized protein composed of 991 amino acids. It features three core helicase subdomains along with an elongated C-terminal region. It shows DNA helicase activity and strand annealing activity and catalyzes the movement of HJs along the DNA helix during DNA replication.^10^

Mutations in three of the RECQ family helicase genes, namely, BLM, WRN, and RECQL4, are linked with genetic syndromes. BLM mutation causes Bloom's syndrome (BS), a rare autosomal recessive disorder, which may lead to an increased risk of cancer, diabetes, or lung diseases.^11^ Several types of mutations in the WRN gene may result in an autosomal recessive progeroid syndrome called Werner syndrome (WS), for which approximately 70 mutations have been identified and studied. These mutations pose a risk of increased cancer susceptibility as well as diabetes mellitus.^12^ RECQL4 mutations have been linked to three clinical diseases: Rothmund–Thomson syndrome (RTS), RAPADILINO syndrome, and Baller–Gerold syndrome (BGS). Rothmund–Thomson syndrome (RTS) has also been linked to an increased risk of osteosarcoma, a type of bone cancer.^13^ Although a human disease has not been directly linked with RECQ1 and RECQ5 mutations, mutations in these helicases may play a role in genomic instability, increased replication stress and cancer.^4^, ^14^ The roles of RECQ helicases in their associated molecular pathways are summarized in Table 1.

**Table 1:** RECQ helicases, associated molecular pathways, and their major biological functions.

Types of RECQ	Molecular Pathways	Major Role	References
RECQ1	Resolution of DNA structures; DNA repair pathways	Resolves and repairs DNA during replication, transcription, or due to any damage	^4^
BLM	Homologous recombination	Suppresses excessive recombination; resolves loops or holliday junctions and repairs DSBs	^5^
WRN	Replication fork stabilization; telomere maintenance	Stabilizes and restarts stalled replication forks and ensures replication fidelity; protects and maintains telomeric sequence and prevents telomere shortening	^7^
RECQL4	DNA replication; mitochondrial DNA maintenance	Initiates DNA replication; ensures accuracy of DNA replication; repairs oxidative damage in mtDNA and maintains its integrity	8, 9
RECQ5	Replication-transcription conflict resolution; homologous recombination	Resolves collisions between replication and transcription machinery; suppresses R-loop formation during transcription; prevents excessive recombination	^10^

This review discusses the primary molecular characteristics and mechanisms of the RECQ-helicase family of proteins. It also delves into the interactions of RECQ helicases with other proteins and the resulting functions of these interactions. Furthermore, the mutations of these helicase genes and the genetic syndromes associated with them.

## 2 BLM helicase: Molecular and enzymatic interactions of BLM protein in bloom's syndrome

BS is a rare genetic disorder that is characterized particularly by short stature, increased sensitivity to the sun, an increased risk of developing certain conditions such as diabetes, and a predisposition to different types of cancer. It follows an autosomal recessive inheritance pattern.^15^ The gaps and breaks in the chromosomes of the cells derived from BS cells increased. Along with such chromosomal abnormalities, a tenfold increase in SCE was observed in these cells. BS is caused by mutations in the BLM gene, which encodes one of the RecQ family DNA helicases.^16^ The BLM protein, as part of a dissolvasome complex, plays a crucial role in resolving double HJ DNA without any genetic exchange. This dissolvasome includes enzymes such as topoisomerase IIIα (TOPIIIα) and RecQ-mediated genome instability (RMI), which consists of two subcomplexes, RMI1 and RMI2, as shown in Fig. 1.^17^

**Fig. 1:**
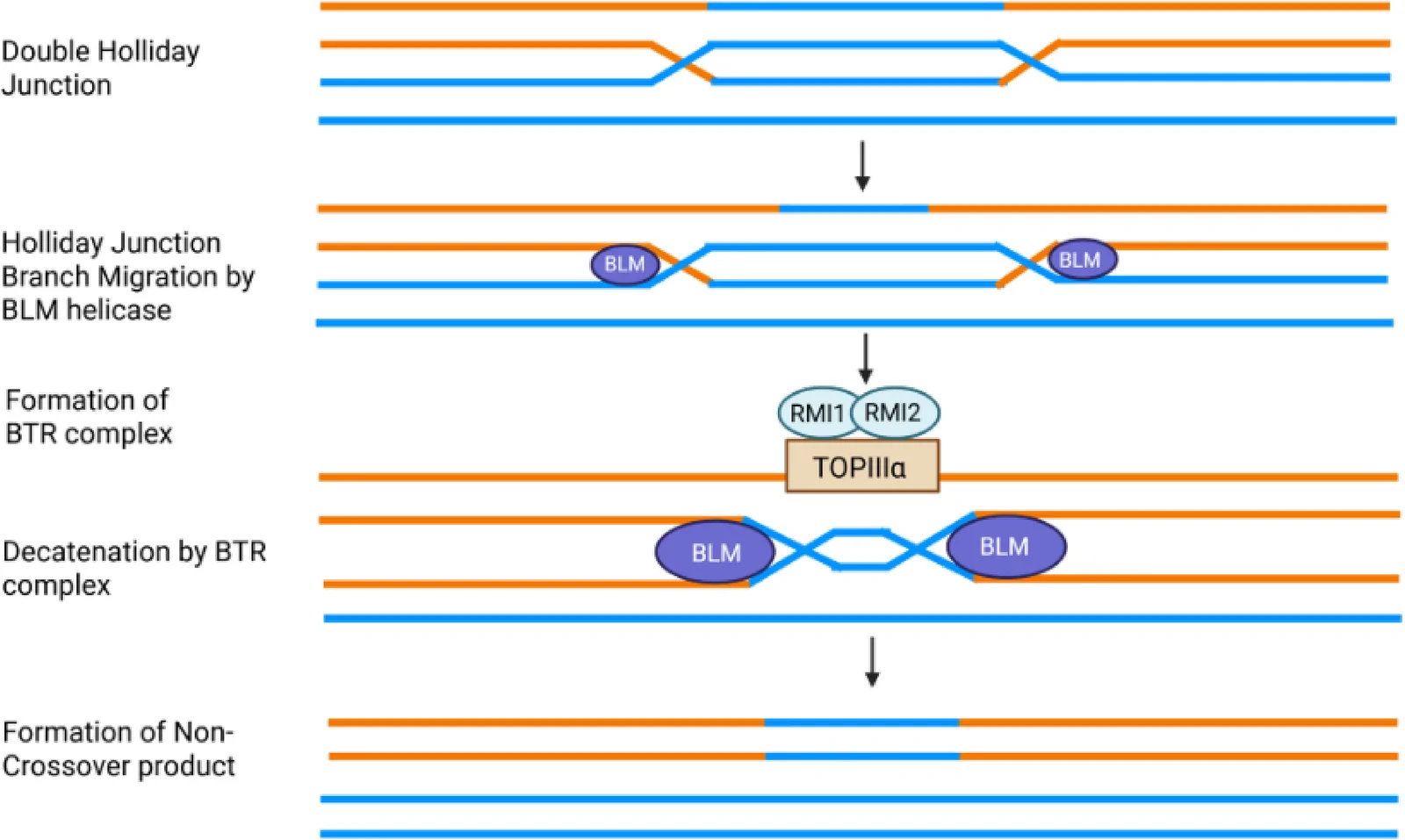
BLM as a part of dissolvasome complex called BTR complex, plays a role in dissolving holliday junctions. *Created with Biorender.*

In vitro experiments conducted by Xu et al. revealed that the RecQ-mediated genome instability (RMI) complex is responsible for the dissolution of homologous recombination (HR) intermediates. The experiments were conducted by inactivating RMI2 in DT40 chicken cells. This cell line has high gene-targeting efficiency and is used for analysing genes involved in DNA repair pathways. The inactivation of RMI2 led to an increase in SCEs, which is an important feature in BS cells. Epistasis analysis also revealed a pathway by which RMI2 and BLM prevent SCEs. The data collected from the experiments revealed that multiple oligonucleotide binding site fold complexes assist the action of the BLM protein in maintaining genomic stability, and RMI is essential for the stability and localization of the BLM complex in cells.^18^ A study conducted by Hoadley KA et al. described the structure and interactions of the RMI core complex. Immunoprecipitation conducted with variants of RMI2 revealed certain key interactions that were responsible for stabilizing the RMI core. RMI1 has oligonucleotide binding sequences that interact with BLM and TOPIIIα. BLM, TOPIIIα, and RMI1 were also coimmunoprecipitated, and major defects in coimmunoprecipitation were detected when RMI2 variants were used. Any disturbance in this interaction within the RMI core led to a very large increase in SCEs. This finding proved that the RMI core was important for stabilizing the BLM dissolvasome, and any variations in RMI caused increased SCE levels, as observed in BS cells.^17^

FANCM refers to Fanconi anemia (FA) complement group M. It is considered a tumor-suppressive gene that codes for a DNA translocase that plays a role in HR and DNA replication. It facilitates the functions of many cellular proteins, such as Caretaker proteins, Bloom's syndrome proteins, Ataxia telangiectasia proteins, RAD3-related proteins, etc. Any changes in these proteins disturb genomic stability.^19^ A study conducted by Deans AJ et al. revealed that the FANCM protein links FA and BS to stalled replication forks and links the components necessary for DNA damage repair. There are two protein–protein interaction complexes, FANCM–Myc–Max 1 (MM1) and Myc–Max 2 (MM2). MM1 interacts with the FA core complex by binding to FA Complement Group F (FANCF). MM2 interacts with RMI and TOPIIIα, which are components of the BS complex. The cells were treated with intercellular cross-linking (ICL)-inducing agents, and the BLM complex interacted with the FA complex to form a supercomplex protein called BRCA1-associated FANCD2 and (RADiation Sensitive 51) RAD51 tumor suppressor complex (BRAFT). Thus, it was suspected that the common symptoms arising in FA and BS could be due to improper BRAFT assembly or activity. Coimmunoprecipitation and purification of FANCM with all the proteins in the BLM and FA complexes revealed that it actively interacted with these proteins. Furthermore, the experiments revealed that the MM1 and MM2 regions independently activated the FA and BS complexes. However, when the interaction of the complexes through FANCM was lost, there was an increase in SCEs and more common phenotypes between FA and BS.^20^ In a study conducted by Basbous & Constantinou, mutations in the FANCM protein caused the affected cells to be more sensitive to DNA cross-linking agents. FANCM is also associated with the BLM complex to promote the dissolution of replication-associated recombinant intermediates. FANCM interacts with RMI and TOPIIIα in the BLM complex. The BLM complex is recruited to DNA damage sites by FANCM. Mutations to FANCM proved that this function of BLM was reduced in mutated cells.^19^ The primary function of RecQ helicases is to relieve replication stress. One way to accomplish this is by preventing the collapse of stalled replication forks. Without this function, stalled replication forks may collapse, leading to severe genomic instability. These pathways are common in telomeric regions. According to a study conducted by Lu et al., the enzyme FANCM facilitates the interaction between the BLM-TOPIIIα-RMI (BTR) complex and the FA enzyme complex. Cancer cells often rely on alternative lengthening of telomeres (ALT), and they exhibit increased levels of replication stress in telomeres. Additionally, they utilize stalled replication forks as templates for break-induced telomere synthesis. Using ALT cell lines, the depletion of FANCM increased ALT activity and break-induced telomere synthesis in the present study. The study concluded that the ability of FANCM to decrease ALT activity requires its association with the BLM-TOPIIIα-RMI (BTR) complex.^22^

In a study conducted by Ellis et al., somatic cells from a person suffering from BS who had undergone excessive mutations at all loci and intragenic recombination within the BLM gene were used and examined. They were treated with bromodeoxyuridine (BrdU), which is used as a marker for tracking DNA replication and cell proliferation. In these cells, there was an increase in the number of interchanges between the homologous chromosomes as well as an increased SCE rate, which provided experimental evidence that the enzyme complex is important for the process of DNA replication. The experiment also revealed that the nascent DNA elongation rate slowed. The above observations revealed a relationship between BLM and topoisomerase II (TOPO II) in human cells, as there was a definite decrease in TOPO II activity in BLM-mutated cells.^23^ A study carried out by Wu et al. revealed two independent domains on the BLM protein that are responsible for the interaction with human topoisomerase IIIα (hTOPIIIα). The biochemical functions of the two proteins are closely related, and the lack of BLM or presence of mutated BLM in BS-affected cells causes hTOPIIIα to be mislocalized in the nucleus. One of the roles of the BLM protein is to bring hTOPIIIα to its site of action, i.e., to bind it to its DNA substrate. The effects of mutations in these protein-coding genes suggest that the BLM-hTOPIIIα protein complex suppresses unnecessary recombination and helps in repairing DNA molecules that have been improperly replicated, the effect of which is observed in BS cells.^24^ Research by Johnson FB et al. demonstrated an association between BLM and TOPIIIα in both somatic and germline cells. Using lymphoblastoid cell extracts, BLM and TOPIIIα were coimmunoprecipitated to show an association in somatic cells. Partial overlap between BLM and TOPIIIα was also observed in meiotic spreads of human testes, suggesting that these proteins have an associated function in germ cells and are present together on meiotic chromosomes.^15^ As previously mentioned, Wu et al. reported that BLM and hTOPIIIα, two TOPIIIα homologues in humans, colocalize in the nucleus of human cells and can be immunoprecipitated from HeLa cell extracts.^24^ Wu et al. further reported that BLM and hTOPIIIα form a protein complex that helps to decaten DNA to break single-stranded DNA (ssDNA) molecules. Any deletions occurring in these 200 amino acids due to mutations in BS cells disable the complex formed between BLM and TOPIIIα. These mutations lead to an increase in SCEs in BS cells, indicating that the interaction of these proteins is important for maintaining the genomic stability of cells.^21^ Chen & Brill reported that a stable TOPIIIα and RMI1 complex could be isolated from yeast cells by purifying and expressing recombinant proteins. This complex highlights the importance of relaxing helical structures within DNA. The isolated RMI1 stimulated TOPIIIα activity. RMI1 binds weakly to ssDNA when it is on its own. However, it also stimulates the binding activity of TOPIIIα with ssDNA. This interaction between RMI1 and TOPIIIα and binding with ssDNA improves the function of RecQ DNA helicases.^25^ Bythell-Douglas, & Deans reported that in BS cells, deletion mutations in the TOPIIIα gene are rare; however, the gene may usually become truncated or unstable, leading to a decrease in the expression of fully functional topoisomerase proteins.^26^ Research conducted by Wang et al. revealed that hTOPIIIα contains two isoforms, one of which contains an N-terminal mitochondrial targeting sequence. Cell fractionation and protease protection assays revealed that some hTOPIIIα is present inside the mitochondria. These findings indicate that mutations in the mitochondrial targeting sequence impair mitochondrial import and function, further affecting mitochondrial DNA (mtDNA) replication and leading to instability.^27^ Despite extensive characterisation of the BLM helicase domain, the N-terminal region of BLM which accounts for more than half of the protein and is predicted to be intrinsically disordered remains poorly understood. A CRISPR/Cas9 deletion study carried out in *Drosophila* identified two conserved N-terminal sub-regions (CR1 and CR2) with distinct functions: deletion of either impaired DSB repair through synthesis-dependent strand annealing and increased mitotic crossovers, but CR2 was additionally critical for embryonic development, while loss of CR1 specifically disrupted meiotic crossover, without affecting chromosome segregation. These findings establish that the intrinsically disordered N-terminal region carries separable regulatory functions beyond those of the catalytic helicase domain, providing a framework for interpreting how non-helicase BLM mutations may contribute to genomic instability and cancer predisposition in BS patients.^28^

## 3 WRN helicase: Molecular and enzymatic interactions of WRN protein in werner's syndrome

Werner gene (WRN) is an important helicase from the RECQ helicase family. A mutated WRN gene may lead to a reduction in the production of WRN protein, causing an autosomal recessive disorder called WS. The WRN gene has exonuclease and helicase domains that perform separate functions. The exonuclease activity domain lies within the first 333 amino acids of the 1432 amino acid chain and is primarily responsible for functions, including resuming stalled replication forks, maintaining DNA fidelity, and fixing DNA mismatch errors, and DNA repair termination of this activity may result in cellular mutations leading to sarcomas. Overall, it helps maintain the genomic stability of the cell.^29^

Another key function of the WRN gene is its involvement in the prevention of telomere shortening. In a study carried out by Chang et al., several generations of WRN-deficient mice presented shorter telomeres in consecutive generations, suggesting the important role of WRN in telomere length maintenance.^30^ Telomerase is another enzyme capable of restoring shortened telomeres by replenishing their length. Cells with a mutated WRN gene struggle to maintain telomeres effectively, even though telomerase is still active. Du et al. demonstrated that the helicase activities of WRN and BLM are responsible for their protective effects on telomeres, and this role becomes increasingly important in cases of telomerase deficiency. These studies revealed that introducing mutated WRN/BLM in telomerase-deficient mice aggravated their premature aging symptoms, along with other phenotypical changes, such as increased chromosomal fusion and accelerated telomere shortening. It was concluded that WRN and BLM are essential for safeguarding telomeres in the absence of telomerase.^31^

On the other hand, the helicase domain of the WRN gene is crucial for binding to diverse DNA substrates and facilitating various DNA metabolic pathways. Some of the DNA secondary structures that WRN can dissolve include X-junctions, D-loops and G-quadruplexes. The WRN gene interacts with several enzymes and proteins to carry out its functions. Some of these include telomeric repeat-binding factor 2 (TRF2), FEN1, DNA polymerase δ, replication protein A (RPA), etc..^32^, ^33^

FEN1 (Flap Endonuclease 1) is a specific endonuclease that plays an important role in DNA replication and repair by cleaving 5′ flaps that are produced during lagging-strand synthesis. WRN and FEN1 interact with each other in human cell nucleoli, and this interaction is crucial for maintaining genomic stability. A study conducted by Brosh et al. revealed that a 144 amino acid domain of WRN acts as the key region for FEN1 cleavage; thus, WRN stimulates the cleavage activity of FEN1. A previous study revealed that these proteins actively interact.^34^

FEN1 plays a crucial role in cleaving the double-flap structure that arises during replication. Precise cleavage allows the removal of excess nucleotides and ensures accurate DNA fragment ligation, thus maintaining the continuity and stability of the newly synthesized DNA strand.^35^, ^36^ However, WRN is not directly involved in the cleavage of the flap and helps and boosts FEN1 to work more efficiently in various ways. It helps in opening the DNA strands, allowing FEN1 access to repair the DNA. The addition of WRN to cells that previously had only FEN1 resulted in the protein working 15 times better at flap cleavage activity. Furthermore, FEN1 and WRN together are responsible for dissolving secondary DNA structures such as the HJ. DNA incision and dissolution were barely detectable when the cells were treated with FEN1 alone, indicating that FEN1 relies on WRN for this activity to occur. WRN can bind to HJ structures and can also physically interact with FEN1. Thus, it acts as a mediator and efficiently stimulates the dissolution of HJs by FEN1.^37^

When the secondary DNA structures cannot be resolved properly during replication, the DNA replisome disassembles, and the replication fork stalls. Damaged replication forks can lead to tumorigenesis and cell death, which are common pathological implications of WS, suggesting that WRN is also linked with stalled replication fork recovery. A study conducted by Zheng et al. revealed that when HeLa cells were introduced to camptothecin (CPT), a topoisomerase inhibitor that blocks DNA replication, the frequency of FEN1-WRN complex formation substantially increased. Additionally, WRN positively stimulated the removal of stalled replication forks by FEN1. Similar stimulation was not observed when the levels of proliferating cell nuclear antigen (PCNA) were increased. These findings suggest that WRN specifically stimulates FEN1 to act on stalled replication forks.^38^ Through coimmunoprecipitation studies, Brosh et al. demonstrated the physical association between WRN and FEN1, establishing their direct physical interaction. They further suggested through certain biochemical assays that WRN enhances the 5′ cleavage activity of FEN1, but this enhancement is dependent on neither WRN exonuclease nor helicase activity. These findings indicate that the noncatalytic domain of WRN mediates its interaction with FEN1.^34^

Another important enzyme actively involved in restoring stalled replication forks is RPA. Various in vitro biochemical assays and colocalization studies by Constantinou et al. revealed that WRN has branch-migration activity that allows it to resolve unnecessary recombinatory intermediates, including HJs. They also illustrated how WRN is recruited from the nucleolus and positioned at stalled replication fork sites, and this recruitment is directly related to its ability to colocalize with replication protein A (RPA).^39^

Noto et al. further identified specific phosphorylation sites within the acidic domain of WRN that are crucial for its association with RPA. WRN binds to the N-terminal domain of RPA1 through this acidic region. Notably, casein kinase 2 (CK2) phosphorylates the acidic domain of WRN, facilitating the formation of the WRN-RPA complex. This interaction is critical for degrading secondary DNA structures, thus facilitating the restoration of replication forks and recovery from replication arrest. The establishment of a stable RPA-WRN complex is required for cellular recovery from stalled replication due to ssDNA gaps. Experimental findings revealed that when the WRN-RPA interaction is compromised, the recovery of replication forks is considerably impaired. Conversely, cells with stable WRN-RPA complexes demonstrate an increased rate of clearance for G-quadruplexes and other secondary DNA structures.^40^, ^41^

In WS patients, owing to the accumulation of secondary DNA structures, an increase in telomeric defects and the compromised ability to restart replication forks, the rates of cell senescence and cell death increase rapidly. These defects arise primarily from mutations in the WRN gene but might be enhanced in the case of improper enzyme activity. The active and precise involvement of enzymes that interact with WRN can enhance its functions and help alleviate symptoms associated with mutations.^42^

Topoisomerases are enzymes that are responsible for preventing DNA from being tangled and relieving the stress caused by coiling and supercoiling during DNA replication.^43^ In several experiments and studies conducted over several years, WS cells exhibited increased sensitivity to topoisomerase inhibitors such as CPT and etoposide (ETOP).^44^

Research by Pichierri et al. demonstrated that cells lacking WRN or containing a mutated version of WRN presented increased chromosomal instability and damage when treated with CPT. Thus, WRN with topoisomerase can collectively dissolve DNA strand breaks and effectively carry out DNA replication. This finding highlights the importance of transcription mechanisms in conjunction with the dual role of topoisomerases and RecQ helicases in preserving chromosomal stability.^45^

Another study by Pichierri et al. revealed that WS cells are increasingly sensitive to topoisomerase inhibitors only at the G2 phase of the cell cycle. Similar behavior was not observed at the G1 phase or S phase. The initial assumption on the basis of this observation was that there might be a defect in the G2 checkpoint response owing to increased sensitivity. However, this assumption was ultimately disproven when experiments revealed no significant differences in mitotic inhibition between the two inhibitors used: amsacrine and VP 16. This finding indicated that the G2 checkpoint functions normally in these cells, as they are still capable of recognizing DNA damage and enforcing mitotic arrest. The lack of differential response to the inhibitors suggests that the increased sensitivity observed in WRN-deficient or WRN-mutated cells is not due to an impairment in the G2 checkpoint but may be linked to other mechanisms. Eventually, it was concluded that the mutation in the WRN gene leading to its lack of proper function with topoisomerase II (TOPO II) caused a defect in the recombinational repair pathway, which operates in the G2 phase before mitosis.^46^

Franchitto et al. studied the role of WRN and TOPO II in the recombination pathway and further experimentally confirmed that the two proteins work together to ensure DNA stability during replication. WS cells treated with the TOPO II inhibitor ICRF187 and X-rays presented increased damage, particularly in the G2 phase. This finding highlights the important role of TOPO II in damage prevention. In WS cells, dysfunction of these proteins leads to DNA instability, chromosomal aberrations and loss of genomic integrity. The study concluded that TOPO II and WRN are crucial for maintaining genomic stability and that their dysfunction might lead to increased damage in WS cells.^47^

Laine et al. carried out various experiments to explain the interactions of WRN and DNA topoisomerase I (TOPO I) and explained their structural and functional associations. TOPO I interacts with two separate sites on the WRN protein, the N- and C-termini. Compared with WRN-mutant WS cells, WS cells presented decreased DNA relaxation activity and subsequently increased chromosomal damage, leading to increased cell death. Another important result of this study was that WRN stimulates the relegation step of TOPO I relaxation. This stimulation by WRN is important, as it prevents the cell from accumulating excess DNA-topoisomerase complexes formed during DNA relaxation.^48^

Building on these findings, Feretti et al., recently demonstrated how WRN targets can be of therapeutic potential by designing a selective allosteric inhibitor HRO761. Inhibition of WRN induced DNA damage, G2 cell-cycle arrest and apoptosis in MSI cancer cells selectively. Furthermore, combining HRO761 with irinotecan, a topoisomerase I inhibitor, enhanced the antitumor activity which strengthened the argument regarding how WRN is important for maintaining genome stability under replication stress.^49^

Recent structural studies have also provided insights into the molecular basis of WRN function. The apo- and DNA-bound crystal structures revealed the domain movements required for helicase activity and identified distinct conformational states targeted by current WRN inhibitors.^50^ Structural studies using cryo-electron microscopy have revealed conformational changes in the WRN helicase core during ATP hydrolysis and DNA unwinding, which reveals additional information regarding the molecular mechanisms underlying DNA substrate recognition and helicase function. These structural findings complement previous biochemical studies describing WRN interactions with proteins such as FEN1, RPA, and topoisomerases.^51^

## 4 RECQL4 helicase: Molecular interactions and associated disorders

RECQL4 is one of the 5 RECQ helicase genes and plays an important role in maintaining genomic homeostasis. The main function of RECQL4 is to preserve genetic material to prevent diseases. It has strong DNA annealing activity and helps to maintain telomere activity. Unlike other RecQ helicases, it does not exhibit helicase activity; however, it facilitates DNA replication initiation and DNA damage repair by assisting in double-strand break (DSB) repair, base excision repair, nucleotide excision repair, DNA crosslink repair, and maintenance of mitochondrial function.^52^ The helicase domains are essential for ATPase and DNA unwinding activity and other regions that control protein–protein interactions. Mutations in RECQL4 lead to certain progeroid syndromes, such as Rothmund–Thomson syndrome (RTS), RAPADILINO syndrome, and Baller–Gerold syndrome (BGS). The human RECQL4 gene encodes an ATP-dependent DNA helicase that contains a conserved helicase domain at the center of the polypeptide.^53^

Kumari et al. created isogenic cell lines in which either the mitochondrial functions of the RECQL4 protein are present or diminished. F_1_F_0_-ATP synthase activity decreased in the absence of RECQL4, and ultimately, mitochondrial integrity was affected. In cells where RECQL4 does not localize to the mitochondria, the membrane potential is reduced, and reactive oxygen species (ROS) levels are increased. A lack of RECQL4 in the mitochondria leads to aerobic glycolysis, increasing the invasive capability of the cells. The rate of mtDNA replication slowed when the RECQL4 gene was mutated in these cell lines. This study revealed that RECQL4 is localized in the mitochondria and acts as an accessory factor during mtDNA replication.^54^ Xu et al. discussed the different molecular and biochemical properties of the various domains of the RECQL4 protein. The molecular role of the N-terminus of the RECQL4 protein was determined when a yeast Sdl2-like domain was found on the protein. The yeast Sdl2 protein is responsible for DNA replication initiation, which indicates that RECQL4 plays a similar role in humans because of the similarity of its N-terminal domain. As the RECQL4 protein also localizes to the mitochondria, it assists in mitochondrial function and biogenesis, and abnormal levels of mtDNA were observed in RECQL4-mutant cells. Analysis of these mutant cell lines could also reveal whether abnormal mitochondrial functions are related to lymphoma or lymphomagenesis. In addition to being involved in DNA synthesis, the RECQL4 protein is involved in DNA damage repair mechanisms. In vitro studies have shown that the RECQL4 protein binds to DNA substrates. Multiple regions in the helicase's N-terminal domain participate in the nucleic acid binding process.^13^ Xu et al. reported that a deletion mutation in the C-terminus of the RECQL4 protein leads to tumorigenic activity. The N-terminus is known for its ability to initiate DNA replication; however, the exact function of the C-terminus in DNA replication is unknown. Any mutation in the C-terminus is prone to cancer, leading to an increase in origin firing, accelerating the transition from G_1_ to S phase, and thus leading to abnormally high DNA content. This study revealed that the C-terminus of the RECQL4 protein has an antagonistic effect on the N-terminus during DNA replication by suppressing its initiation. Oncogenic mutations impair this antagonistic function.^55^

RTS is a rare genetic disorder associated with an increased risk of cancer, resulting from mutations in the RECQL4 gene. Patients with RTS exhibit aneuploidy, characterized by the gain or loss of a chromosome. Additionally, chromosome mosaicism and the presence of isochromosomes on chromosomes 2, 7, and 8 have been observed in individuals with RECQL4 mutations.^56^ Lindor et al. studied a patient with RTS and identified a compound heterozygous mutation in the RECQL4 gene. This consisted of a single-base deletion in exon 9 causing a frameshift and the formation of an early termination codon as well as a base change at the 3′ splice site of exon 8, leading to a deletion within the helicase domain.^57^ Macris et al. expressed the human RECQL4 protein in *E. coli* and reported that RECQL4 has ATPase activity activated by DNA, especially ssDNA. It was also determined that RECQL4 shows DNA annealing activity inhibited by replication protein A (RPA), which is a ssDNA-binding protein. Compared with other RecQ helicase proteins, which primarily show DNA unwinding activity during replication, cells deficient in the RECQL4 protein present increased genomic instability; thus, this enzyme may have a DNA repair function.^58^ A sequencing analysis carried out by Sznajer et al. revealed that the RECQL4 mutated gene in RTS had a splice site mutation and a nucleotide substitution in exon 12. This study demonstrated how mutations in RECQL4 cause RTS as well as RAPADILINO syndrome, leading to overlapping phenotypes in these disorders, and some are even observed in BGS. The truncating mutation is revealed to be a missplicing error during transcription, which suggests that these mutations lead to the formation of a nonfunctional protein, explaining the symptoms and leading to an increased risk of osteosarcoma in patients with RECQL4 mutations.^59^ Fang et al. demonstrated the interaction between RECQL4 and the enzyme Aurora B kinase (AURKB). RECQL4 physically and functionally interacts with AURKB, stabilizing its expression by inhibiting its ubiquitination. The N-terminal domain of RECQL4 physically interacts with the catalytic domain of AURKB. When RECQL4 expression is suppressed, the expression of AURKB is also reduced, which leads to mitotic instability. It was concluded that the RECQL4-AURKB enzyme interaction is important for cell proliferation and cell cycle regulation and that any disturbances in this interaction may lead to abnormal cell division and abnormalities in the cells.^60^ Castillo et al. introduced an ATP-binding-deficient knock-in mutation (K525A variant) in mice. This mutation specifically reduces ATP-dependent helicase activity. This analysis was performed to determine the functions of the RECQL4 helicase and its activity in a biological system. The K525A variant can bind DNA but cannot hydrolyse ATP or unwind DNA substrates. The results revealed that the mutant protein did not function when ATP was present and displayed a loss of helicase activity.^61^ In the study conducted by Jewell et al., induced pluripotent stem cells (iPSCs) were generated from patients suffering from Type 2 RTS to understand the pathology underlying the relationship between RTS and increased risk of osteosarcoma. The RTS iPSC-derived osteoblasts presented defects in osteogenic differentiation and increased tumorigenic ability in in vitro experiments. Further analysis revealed a decrease in bone morphogenesis and abnormally upregulated mitochondrial respiratory complex I gene expression, increased oxidative phosphorylation (OXPHOS), and increased ATP production. When mitochondrial respiratory complex I (MRC 1) was inhibited, the proliferation of RTS osteoblasts was suppressed. Therefore, the study concluded that MRC-1 could be a possible clinical treatment strategy for RTS-associated osteosarcoma.^62^ As discussed by Balajee et al., an increased occurrence of osteosarcoma is observed in RTS patients with RECQL4 mutations, and there is an increase in the expression of RECQL4 in sporadic cancers, especially osteosarcoma, suggesting that aneuploidy of RECQL4 is linked to an increased risk of cancer. Therefore, identifying potential ways to use RECQL4 as a novel target for cancer therapeutics is important. RecQL4 suppression in nonmalignant epithelial cells was observed to reduce proliferation by only 20% compared with more than 50% in prostate cancer cells. This signifies that replication dependency on RECQL4 is greater for cancer cells than for nonmalignant-type cells. It was also speculated on the basis of different experimental results that RECQL4 maintains the cell cycle and mitotic integrity of cancer cells while protecting them from endogenous and exogenous DNA damage. RECQL4 integrates with p53, and telomerase activity contributes to the increased replicative capacity of cells. Therefore, the use of this strategy to regulate the RECQL4 signalling pathway could be an effective therapeutic strategy.^63^ A study by Hong et al. revealed an additional mechanism by which elevated RECQL4 expression promotes tumor progression in hepatocellular carcinoma (HCC): by efficiently repairing radiation-induced DNA DSBs, RECQL4 reduces the release of cytosolic DNA fragments that would otherwise activate the cGAS-STING innate immune pathway in tumor-infiltrating dendritic cells. This suppresses dendritic cell and CD8+ T cell recruitment into the tumor microenvironment, effectively blunting the anti-tumor immune response triggered by radiotherapy. Knockdown of RECQL4 or deletion of STING in dendritic cells confirmed the mechanistic link, and high RECQL4 expression in HCC patients correlated significantly with poor survival. These findings extend the oncogenic role of RECQL4 beyond DNA repair and cell cycle regulation, and position RECQL4 as a potential target in combined radiotherapy-immunotherapy strategies for HCC.^64^ RECQL4 overexpression has also been implicated in lung adenocarcinoma, where elevated RECQL4 levels in clinical tumor tissues are associated with poor prognosis and adverse clinicopathological features. Gain- and loss-of-function experiments demonstrated that RECQL4 promotes the proliferation, migration, and invasion of cancer cells through activation of the NF-κB signalling pathway, revealing a signalling axis distinct from the AKT-mediated pathway described in gastric cancer. This expanding range of downstream pathways through which RECQL4 supports tumor progression underscores the context-dependent molecular roles of this helicase across different cancer types.^65^ Marmolejo et al. reported that multiple variations in the RECQL4 gene can cause patients to develop osteosarcoma. The mutations are biallelic variations in the RECQL4 gene, and along with RECQL4, anaphase promoting complex subunit 1 (ANAPC1) mutations can also lead to this syndrome; however, they are not related to osteosarcoma. The interaction between RECQL4 and ANAPC1 ensures the regulation of mitotic checkpoints. These genetic alterations cause chromosomal instability, which may lead to a predisposition to various types of cancer and increase the risk of chemotherapy-induced toxicity.^66^ In RTS, RECQL4 can interact with poly (ADP–ribose) polymerase 1 (PARP1), a nuclear enzyme that also plays a role in maintaining genomic stability, by participating in DNA repair and signalling pathways. It is possible that the carboxy (C)-terminus of RECQL4 might be a substrate for PARP1, which is suggestive of a relationship between the two enzymes in response to oxidative stress.^67^

RAPADILINO syndrome is a genetically inherited disorder characterized by phenotypes such as short stature, radial ray abnormalities, other congenital malformations, and infantile diarrhea. It is inherited in an autosomal recessive pattern. Unlike RTS, there is no significant risk of cancer. There are four mutations in RECQL4, including a deletion in exon 7 and three other nonsense mutations in exons 5, 18, and 19.^68^ In accordance with the methods of Fradin et al., DNA samples from patients' blood were taken and amplified via polymerase chain reaction (PCR). Sequencing of RECQL4 revealed the presence of a truncating mutation and a missense mutation, both of which are located outside the helicase domain of the protein. However, despite having a truncating mutation, the patient had not yet developed any tumors.^69^ Although it is known that RAPADILINO shares common mutations and symptoms with RTS, there is no definitive molecular pathway that explains why RTS patients are more prone to developing tumors. Yin et al. reported that RECQL4 physically interacts with ubiquitin ligase proteins, namely, UBR1 and UBR2. These findings indicate that the RECQL4 modification and degradation mechanism may be strongly regulated by ubiquitination. Hence, mutations in not only the RECQL4 helicase gene but also its posttranslational regulators lead to syndromes. However, it is also important to establish the exact molecular alterations in the interactions of these proteins to identify the differences in the phenotypes of these syndromes, despite being caused by the same gene.^70^

Baller–Gerold syndrome (BGS) is a rare autosomal recessive disorder characterized by radial aplasia/hypoplasia and craniosynostosis caused by biallelic RECQL4 mutations. Some patients with BGS present with poikiloderma, which is a distinct characteristic of RTS. In one patient, compound heterozygosity was observed, as there were a missense mutation and a frameshift mutation in exon 9. In addition, there was a homozygous splice site mutation, and both patients had craniosynostosis and radial defects in common along with poikiloderma. These observations clearly showed that BGS belongs to the same clinical spectrum as RTS and RAPADILINO syndromes, along with a significant molecular and phenotypic overlap caused by RECQL4 mutations.^71^ Fig. 2 illustrates the common symptoms of RECQ disorders.

**Fig. 2:**
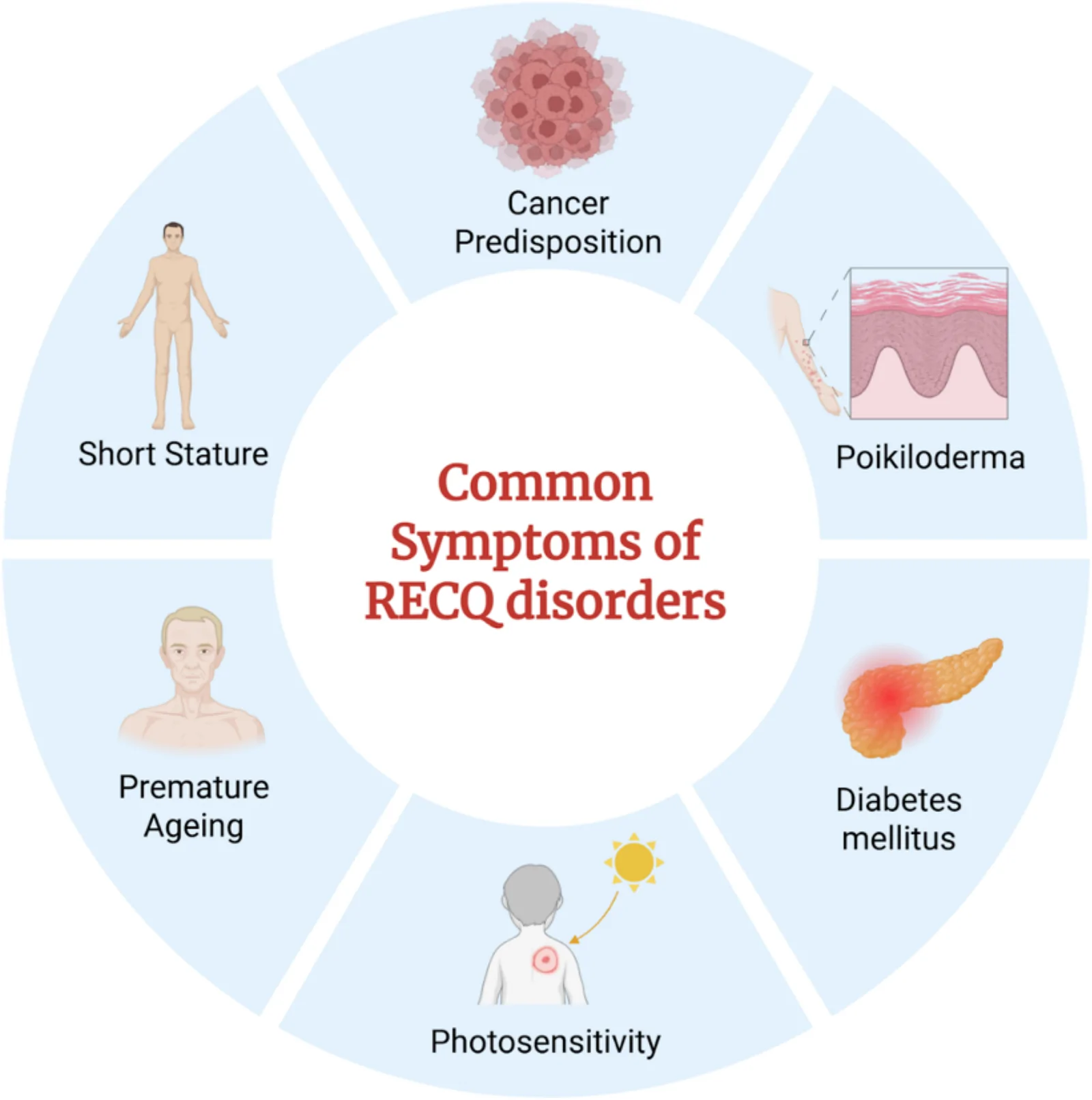
Visual depiction of common symptoms of BLM, WRN and RECQL4- associated genetic disorders. *Created with Biorender.*

## 5 RecQ1 helicase: Molecular interactions and functional significance

RECQ1, also known as RECQL1 or RECQL, was the first RecQ homologue identified in humans. Although RECQ1 is not yet associated with any well-defined syndromes, mutations in these genes have been shown to cause several chromosomal aberrations and genomic instability. A lack of RECQ1 or mutations in RECQ1 hinder the normal activities of strand separation and annealing as well as HJ resolution. This leads to increased genomic instability.^72^

The truncated catalytic core of the human RECQ1 helicase has a beta-hairpin structure. This beta-hairpin is located in the winged helix (WH) domain of RECQ1. This structure plays an important role in the resolution of HJs and helps increase the efficiency of DNA unwinding. The beta hairpin is also necessary to form RECQ1 dimers (RECQ1 49–616) and tetramers, which help in DNA unwinding. Tetramers also play important roles in HJ resolution and strand annealing.^73^

RECQ1 plays a key role in DNA repair and resolution during transcription, replication, and in response to damage. To carry out these functions, it interacts with various proteins. Studies on the association of RECQ1 with mismatch repair factors revealed through coimmunoprecipitation that RECQ1 physically interacts with human exonuclease 1 (EXO1). RECQ1 enhances EXO1 incision activity, and notably, even a helicase-dead RECQ1 mutant was able to stimulate this activity. Furthermore, this stimulation occurred independently of ATP, indicating that the helicase function of RECQ1 is not essential for activating EXO1. These findings demonstrate that RECQ1 and exonuclease 1 (EXO1) interact both physically and functionally, with RECQ1 promoting EXO1 nuclease activity through a helicase-independent mechanism.^74^

RECQ1 also interacts with FEN1, as demonstrated by Sami et al., who showed through coimmunoprecipitation experiments that RECQ1 and FEN1 physically associate in cells and that this protein–protein association is DNA independent, implying a direct, functional correlation between these proteins. RECQ1 was shown to enhance the endonucleolytic cleavage of 5′-flap DNA substrates. Both RECQ1 and FEN1 were consistently present at telomeres, with increased binding to telomeric chromatin being observed after DNA damage. Chromatin immunoprecipitation revealed that the absence of RECQ1 led to reduced FEN1 binding at telomeres and diminished its activity, highlighting the cooperative role of these two proteins and the dependency of FEN1 on RECQ1 for telomeric repair.^75^

Several experiments conducted by Cybulski et al. and Sun et al. revealed that certain mutations associated with RECQ1 increased the risk of breast cancer. Cybulski et al. reported deleterious germline mutations in breast cancer patients. These mutant RECQ1 proteins presented reduced helicase activity, implying a lack of proper function, which is essential for DNA repair. Sun et al. carried out exome sequencing of breast cancer patients followed by large-scale genotyping and identified five different deleterious RECQ1 mutations of different types, including missense, nonsense and frameshift mutations. The mutated proteins presented defective DNA repair.^76^, ^77^ Recently, Abu-Libdeh et al. linked RECQ1 mutations to genomic instability syndrome, which they named RECql ONe (RECON) syndrome. A homozygous biallelic missense mutation (pAla459Ser) was associated with striking early aging symptoms and other physiological disorder characteristics, including skin photosensitivity, xeroderma, etc. The clinical phenotypes of families affected by these symptoms were studied, and through exome sequencing, this mutation was identified. The p.A459S amino acid substitution in the ZBD of RECQ1 led to compromised DNA unwinding activity, which was identified as the root of this genomic instability. The restoration of the replication fork performed by the mutated enzyme is significantly lower. The DNA substrate binding activity is also compromised. These mutated cells showed a reduced ability to replicate in the presence of topoisomerase inhibitors such as CPT and ETOP, leading to the conclusion that cells of patients with RECON syndrome have a decreased capacity to replicate when faced with abortive topoisomerase lesions.^78^

## 6 RECQ5 helicase: Molecular interactome and functional implications

RECQ5 codes for helicase enzymes, which are involved in DNA replication, transcription, HR, and repair. It has a helicase and ATPase domain as well as protein-interacting regions. As it is tightly combined with HR, it performs an important function in stabilizing the genome, and any mutation in the RECQ5 gene can lead to chromosomal or genomic rearrangements.^79^

A study by Aygün et al. revealed that RECQ5 is an RNA polymerase II (RNAPII)-associated protein. A subunit of RNAPII mediates this interaction between two proteins called retinol binding protein 1 (RBP1). This interaction suggests a role for the RECQ5 helicase in transcription and maintaining genomic stability. Moreover, the RECQ5-mutant or RECQ5-knockout mouse models presented genomic instability and increased incidence of cancer. Chromatin-associated proteins were purified to study all the protein interactions of RNAPII. RECQ5 was tagged with FLAG protein, which was the source of purification, suggesting that the cells stably expressed human RECQ5 and that it interacted with RNAPII.^80^ The structural basis for this RECQ5-RNAPII relationship has now been resolved at molecular resolution. A cryo-EM study has reported structures of RECQ5 bound to transcription elongation complexes, revealing that a specific α-helix of RECQ5 inserts into the RNAPII active-site cleft to slow transcription elongation, a “transcription braking” mechanism that reduces the rate of progression and thereby limits the accumulation of replication-transcription conflicts. Crucially, the transcription-coupled DNA repair (TCR) complex was shown to override this brake through its translocase activity, providing a regulated on/off switch for transcription elongation speed.^81^ Urban et al. reported that RECQ5 plays a role in resolving conflicts in the processes of replication and transcription. This inconsistency between the two molecular mechanisms contributes to genomic instability. It has also been suggested that RECQ5 associates with RNA polymerase I (RNAPI) and promotes the stability of ribosomal DNA. It also interacts with transcription complexes and prevents stalled replication fork formation in the genes transcribed by RNAPI and RNAPII, which further proves that RECQ5 acts at replication and transcription collision sites. When RECQ5 is deficient in cells, RAD18 accumulates at replication-transcription interference sites. Moreover, RECQ5 depletion also causes unresolved replication intermediates to accumulate at the sites of replication–transcription collisions.^82^ A study by Andrs et al. investigated the mechanism and roles of RECQ5 in DSB repair. The RECQ5 helicase maintains genomic stability by carrying out HR to repair DSBs. HR is carried out by disrupting RAD51 nucleoprotein filaments. RAD51 filaments initiate the process of double-strand break (DSB) repair, which ultimately forms a displacement loop that leads to the formation of a crossover. RECQ5 acts as a regulator of this potentially hazardous crossover formation in HR, as it does not allow the initiation of HR by disrupting the formation of RAD51 presynaptic filaments. Additionally, when RECQ5 levels are reduced, there is an increase in crossing, which leads to increased sister chromatid exchange (SCE), leading to genomic instability and a predisposition to cancer. Moreover, when RECQ5 expression is upregulated, HR has an inhibitory effect, and DSB repair is carried out by increasing the degree of noncrossing over (NCO) of chromatids.^14^

Li et al. reported that the K391 and K436 residues on the DNA topoisomerase I (TOPO1) protein are SUMOylated, which is a posttranslational modification required to tag proteins for transcription and other cellular processes. This process of SUMOylation is carried out by the protein inhibitor of activated STAT 1- serine/arginine-rich splicing factor (PIAS1-SRSF1) E3 ligase complex in chromatin containing active RNAPII. This modification is necessary for TOPO I to bind to RNAPII and carry out its function during transcription, i.e., reducing the formation of R-loops to prevent any genomic instability. RECQ5 mediates this entire process by increasing the association of the PIAS1, SRSF1, and TOPO I proteins. In vitro SUMOylation assays were conducted to study the levels of proteins, PIAS1, SRSF1, and TOPO I. Further evidence was given that any defect in RECQ5 led to improper TOPO I SUMOylation, which in turn led to an increase in the formation of R-loops and DSBs, causing genomic instability.^83^ Hamadeh & Lansdorp reported that mice deficient in RECQ5 had greater chances of developing cancer, and human cells deficient in RECQ5 presented chromosomal instability and increased SCE. The collision of replication and transcription events can result in intermediate DNA repair structures, which can be resolved by RECQ5. Additionally, during replication, many stressors cause stalling of the replication fork. RECQ5 plays a role in protecting the process of replication from these stresses, as the cells deficient in RECQ5 were more sensitive to the TOPO I inhibitor camptothecin, which may lead to improper replication and an increase in genomic instability. RECQ5 plays an important role as a regulator in replication and transcription mechanisms and in repairing intermediate structures that may stall these processes.^10^

HR is one of the DNA DSB repair pathways. Alpha thalassemia/Mental Retardation Syndrome X-Linked (ATRX), which is a chromatin remodeller, initiates an alternative subpathway for HR that may form crossovers in the DNA. Synthesis-dependent strand annealing (SDSA) depends on RECQ5, another HR subpathway that prevents the formation of crossovers and occurs predominantly in mammalian cells.^84^ A study conducted by Elbakry et al. further characterized the role of RECQ5 in HR. In the presence of any mutations in the ATPase domain (RECQ5-ATP) or the PCNA-interacting region (RECQ5-PCNA), the RECQ5 helicase fails to repair DSBs. Similarly, ATRX-PCNA mutations also fail to repair DSBs. The above study suggested that the efficiency of HR subpathways depends on the interaction of alpha thalassemia/mental retardation syndrome X-linked (ATRX) and RECQ5 with PCNA.^85^ The RECQ helicases and their associated protein interactions are presented in Table 2.

**Table 2:** RECQ helicases, their interacting proteins, and associated biological functions.

Types of RECQ	Interacting proteins	Associated function	References
BLM	1. TOPIIIα 2. RMI complex- RMI1 and RMI2 3. FANCM 4. TOPO II	1. Dissolution of double holliday junctions 2.Stabilization of the BTR complex 3.Prevents excessive SCEs 4.Resolves DNA supercoiling	^17^ 17, 18 19, 20, 21, 22 ^43^
WRN	1.FEN1 2.RPA 3.Topoisomerase	1.Enhanced flap cleavage activity, resolution of secondary DNA structures and recovery of stalled replication forks 2.Cleavage of secondary DNA structures, processing ssDNA gaps 3.Recombinational repair, Prevention of DNA damage	34, 35, 36 39, 40, 41 44, 45
RECQL4	1.RPA 2.AURKB 3.ANAPC1 4.PARP1	1.Initiation of DNA replication 2.Cell cycle regulation 3. Ensuring mitotic checkpoints 4. Facilitates DNA damage repair	^58^ ^60^ ^66^ ^67^
RECQ1	1. EXO1 2. FEN1	1. Nuclease and incision activity 2. Facilitates DNA repair damage especially at telomeres	^74^ ^75^
RECQ5	1. NAPII 2. NAPI 3. RAD51 4. TOPO1, PIAS1, SRSF1 5. PCNA and ATRX	1. Regulates Transcription and resolves replication-transcription conflicts 2. Promotes the stability of ribosomal DNA 3. Resolves DSBs and HJs 4. Prevents formation of R-loops and repairs DSBs 5. Repairs DSBs	80, 81 ^82^ ^14^ ^83^ ^85^

## 7 Discussion

RecQ helicases are a family of proteins that play important roles in DSB repair and preventing genomic instability. Defects in these proteins caused by various gene mutations can cause specific genetic disorders and lead to tumorigenesis.^1^ The BLM gene codes for the BLM helicase, and mutations in this gene cause BS.^11^, ^23^ BS cells show an increase in chromosome breaks, gaps and SCEs, which establish that the BLM helicase functions in maintaining genomic stability.^87^ The BTR complex, comprising BLM, TOPIIIα, and the RecQ-mediated genome instability proteins RMI1 and RMI2, plays a crucial role in resolving HR intermediates and suppressing excessive recombination.^5^ FA complement group M (FANCM) is another protein that links BLM with the FA pathway, forming a complex that repairs DNA damage and maintains telomere stability. Disruptions in these complexes lead to an increase in SCEs and genomic instability.^19^, ^20^ The WRN gene, which encodes the WRN helicase, is responsible for causing WS in the case of mutations. The WRN gene is primarily responsible for maintaining the length of telomeres as well as dissolving secondary structures.^6^, ^7^ It interacts with several proteins and facilitates various functions. WRN assists FEN1 in flap cleavage, colocalizes with RPA at stalled replication forks, resolves them, and interacts with topoisomerases to reduce replication stress.^34^ Mutations in the WRN gene may lead to chromosomal instability and unresolved replication forks due to the limited interaction of the mutated gene with these proteins. WRN deficiency may also lead to progressive telomere shortening, resulting in increased cell senescence and cancer.^31^ RECQL4 is an important helicase involved in genomic stability, the initiation of DNA replication, DNA damage repair and the maintenance of mitochondrial function. Mutations in the RECQL4 gene lead to the progeroid syndromes Rothmund-Thomson syndrome (RTS), BGS and RAPADILINO.^9^, ^13^ RTS has been specifically linked to osteosarcoma; however, BGS and RAPADILINO have relatively mild effects on cancer.^56^, ^62^ RECQL4 interacts with proteins such as AURKB, PARP1, and telomerase, contributing to replication pathway stability and playing a key role in tumor suppression.^60^, ^67^ Mutations in the RECQL4 gene impair ATPase, helicase and DNA repair pathways, leading to instability in the genome, mitochondrial dysfunction and increased oxidative stress.^61^, ^62^ RECQ1 is responsible for DNA unwinding, strand annealing and HJ resolution.^3^, ^4^ Its beta-hairpin structure and zinc-binding domain (ZBD) are essential for helicase activity.^73^ Mutations in RECQ1, particularly those in the ZBD, are linked to a syndrome referred to as RECON syndrome, which leads to cells having impaired replication capacity and a reduced ability to recover from replication stress.^78^ Recent studies have also suggested that RECQ1 can act as a tumor suppressor gene and that mutations may be linked to risks associated with breast cancer.^76^, ^77^ RECQ5 is responsible for maintaining genomic stability by regulating HR, transcription and DNA repair pathways.^14^ It interacts with RNA polymerase II (RNAPII) with the help of RBP1 and resolves replication–transcription conflicts.^80^, ^82^ It also facilitates the SUMOylation of DNA topoisomerase I (TOPO1), which prevents replication fork stalling and resolves R-loops.^83^ Disruption of RAD51 filaments suppresses crossover formation in HR, thereby promoting noncrossover repair. Its interaction with PCNA is crucial for HR efficiency, functioning alongside alpha thalassemia/mental retardation syndrome X-linked (ATRX) to regulate HR subpathways.^84^, ^85^ Thus, RECQ5 deficiency leads to genomic instability, increased SCE, and increased cancer susceptibility.

In a series of studies conducted via mass spectrometry, several posttranslational modifications, such as ubiquitination and acetylation, have been observed on RecQ helicases.^86^ However, the exact relevant functions of these alterations are still in question and can be studied further. Importantly, individuals suffering from BS due to BLM gene mutations are more predisposed to cancer. Although the function of the BLM gene in maintaining genome stability is known, the exact reasons and molecular mechanisms underlying the risk of cancer need to be studied further to be completely understood.^87^ WRN helicase, which arises from the accumulation of short repetitive DNA sequences known as microsatellites, has been identified as a synthetic lethal target in microsatellite instability (MSI) cancers. MSI occurs due to defects in the DNA mismatch repair system.^88^ WRN protein levels have been reported to decrease in cells treated with CPT, which is an anticancer drug.^89^ These findings suggest that cancers with MSIs can serve as potential targets for CPT therapy for WRN-specific tumors. This finding also highlights the possibility that WRN can act as a potential drug target for cancer. Some evidence indicates the involvement of RecQ helicases in the DNA replication checkpoint; however, the exact molecular mechanisms involved in checkpoint signalling require further research.^2^ Since RECQL4 is the only known RecQ helicase that can localize to the mitochondria, further study of its functions in the mitochondrial compartment and determination of the factors that determine whether RECQL4 must localize to telomeres or mitochondria are important. RECQL4 may have a possible signalling function between the two compartments.^9^ Sun et al. reported that the RECQ1 gene is directly associated with a predisposition to breast cancer.^77^ In addition to its important role in maintaining genomic stability, RECQ1 acts as a tumor suppressor gene, and its mutations are linked with hereditary breast cancer. More research is needed to assess the risks of various RECQ1 variants and their effects in this area.^3^ Recent studies have suggested that RECQ5 variants may be potential drug targets, especially in gastric cancers and osteosarcoma.^90^, ^91^ The overexpression of RECQ5 leads to the progression of tumors in urothelial bladder carcinoma.^92^ These observations provide an avenue for developing potential therapeutic strategies using RECQ5 as a target. Some of these dependencies are already being developed into practical treatment strategies. Based on its identification as a synthetic lethal target in MSI cancers, WRN has recently been targeted with a covalent, allosteric small-molecule inhibitor that selectively kills MSI-high cancer cells while sparing microsatellite-stable cells. At the same time, structural studies are helping guide the design of more selective helicase inhibitors.^51^, ^93^ RECQ1 overexpression has also been linked to resistance to the PARP inhibitor olaparib in ovarian cancer, and reducing RECQ1 levels restores sensitivity to olaparib in resistant cells. This suggests that inhibiting RECQ1 could be used together with PARP inhibitors to overcome drug resistance, rather than as a treatment on its own.^94^, ^95^ In contrast, no selective RECQL4 inhibitor is currently available, so RECQL4 expression is instead being studied as a prognostic and predictive biomarker, for example to identify patients who are more likely to respond to cisplatin-based chemotherapy.^96^ Together, these examples suggest that achieving selectivity across the highly conserved RECQ helicase core, and combining helicase inhibitors with existing DNA damage response-targeted therapies, rather than using helicase inhibition alone, will be key to turning these molecular interactions into effective clinical treatments.

As further research progresses, RECQ helicases have proven to be potential therapeutic targets because of their roles in DNA repair and genome maintenance. Inhibiting or promoting their function by controlling their gene expression could lead to novel treatment strategies for genetic disorders as well as cancer.

## Human and animal rights and informed consent

This article does not contain any studies with human or animal subjects performed by any of the author.

## CRediT authorship contribution statement

**Yashaswini Reddy:** Writing – review & editing, Writing – original draft. **Gargi Joshi:** Writing – review & editing, Writing – original draft. **Swechha Mahapatra:** Writing – review & editing, Writing – original draft. **Ipshita Das:** Writing – review & editing. **Babitha Kampa Sundara:** Supervision, Conceptualization.

## Declaration of generative AI and AI-assisted technologies in the writing process

During the preparation of this work the author(s) used Quillbot to Paraphrase. After using this tool/service, the author(s) reviewed and edited the content as needed and take(s) full responsibility for the content of the publication.

## Funding declaration

Authors thank Intramural funding (Grant ID: MAHE/CDS/PHD/IMF/2023), MAHE, Manipal for financial support. Authors also thank MRB Seed grant (Grant ID: DOR/MRB/2023/SG-03), MAHE, Manipal for funding. Y.R. thank Manipal Academy of Higher Education for Dr. TMA Pai Ph.D. fellowship.

## Declaration of competing interest

The authors declare that they have no known competing financial interests or personal relationships that could have appeared to influence the work reported in this paper.
